# Peroxynitrite Exposure of CXCL12 Impairs Monocyte, Lymphocyte and Endothelial Cell Chemotaxis, Lymphocyte Extravasation *in vivo* and Anti-HIV-1 Activity

**DOI:** 10.3389/fimmu.2018.01933

**Published:** 2018-08-28

**Authors:** Rik Janssens, Daiane Boff, Pieter Ruytinx, Anneleen Mortier, Vincent Vanheule, Olav Larsen, Viktorija Daugvilaite, Mette M. Rosenkilde, Sam Noppen, Sandra Liekens, Dominique Schols, Ingrid De Meester, Ghislain Opdenakker, Sofie Struyf, Mauro M. Teixeira, Flávio A. Amaral, Paul Proost

**Affiliations:** ^1^Laboratory of Molecular Immunology, Department of Microbiology and Immunology, Rega Institute for Medical Research, KU Leuven, University of Leuven, Leuven, Belgium; ^2^Departamento de Bioquímica e Imunologia, Instituto de Ciencias Biologicas, Universidade Federal de Minas Gerais, Belo Horizonte, Brazil; ^3^Laboratory for Molecular Pharmacology, Department of Biomedical Sciences, Faculty of Health and Medical Sciences, The Panum Institute, University of Copenhagen, Copenhagen, Denmark; ^4^Laboratory of Virology and Chemotherapy, Department of Microbiology and Immunology, Rega Institute for Medical Research, KU Leuven, University of Leuven, Leuven, Belgium; ^5^Laboratory of Medical Biochemistry, Department of Pharmaceutical Sciences, University of Antwerp, Wilrijk, Belgium; ^6^Laboratory of Immunobiology, Department of Microbiology and Immunology, Rega Institute for Medical Research, KU Leuven, University of Leuven, Leuven, Belgium

**Keywords:** chemokine, post-translational modification, inflammation, nitration, lymphocyte migration

## Abstract

CXCL12 is a chemotactic cytokine that attracts many different cell types for homeostasis and during inflammation. Under stress conditions, macrophages and granulocytes produce factors such as peroxynitrite as a consequence of their oxidative response. After short incubations of CXCL12 with peroxynitrite, the gradual nitration of Tyr7, Tyr61, or both Tyr7 and Tyr61 was demonstrated with the use of mass spectrometry, whereas longer incubations caused CXCL12 degradation. Native CXCL12 and the nitrated forms, [3-NT^61^]CXCL12 and [3-NT^7/61^]CXCL12, were chemically synthesized to evaluate the effects of Tyr nitration on the biological activity of CXCL12. All CXCL12 forms had a similar binding affinity for heparin, the G protein-coupled chemokine receptor CXCR4 and the atypical chemokine receptor ACKR3. However, nitration significantly enhanced the affinity of CXCL12 for chondroitin sulfate. Internalization of CXCR4 and β-arrestin 2 recruitment to CXCR4 was significantly reduced for [3-NT^7/61^]CXCL12 compared to CXCL12, whereas β-arrestin 2 recruitment to ACKR3 was similar for all CXCL12 variants. [3-NT^7/61^]CXCL12 was weaker in calcium signaling assays and in i*n vitro* chemotaxis assays with monocytes, lymphocytes and endothelial cells. Surprisingly, nitration of Tyr61, but not Tyr7, partially protected CXCL12 against cleavage by the specific serine protease CD26. *In vivo*, the effects were more pronounced compared to native CXCL12. Nitration of any Tyr residue drastically lowered lymphocyte extravasation to joints compared to native CXCL12. Finally, the anti-HIV-1 activity of [3-NT^7^]CXCL12 and [3-NT^7/61^]CXCL12 was reduced, whereas CXCL12 and [3-NT^61^]CXCL12 were equally potent. In conclusion, nitration of CXCL12 occurs readily upon contact with peroxynitrite and specifically nitration of Tyr7 fully reduces its *in vitro* and *in vivo* biological activities.

## Introduction

CXCL12 is a homeostatic CXC chemokine that exists in six splice variants and is expressed in most tissues (1). Initially, CXCL12 was discovered as a pre-B cell growth factor of crucial importance for lymphopoiesis and embryogenesis and was shown to be a co-stimulator for CD4^+^ T cell activation (2, 3). Regarding its chemotactic activity, CXCL12 was purified from its main source, the bone marrow stroma, based on its ability to attract lymphocytes and monocytes (4). In the bone marrow, CXCL12 prevents the release of hematopoietic progenitor cells into the circulation (5). Furthermore, CXCL12 is an angiogenic chemokine and attracts endothelial progenitor cells in response to hypoxia (6). CXCL12 activity has also been shown in several inflammatory conditions, such as rheumatoid arthritis, inflammatory bowel disease and cancer growth and metastasis (3, 7, 8).

To fulfill these functions, CXCL12 activates its 7-transmembrane spanning (7TM) G protein-coupled receptor (GPCR) CXCR4 and atypical chemokine receptor ACKR3 (9–11). As is the case for CXCL12, CXCR4 knock-out mice die perinatally due to defects in lymphopoiesis and neurogenesis (2, 12). ACKR3 knock-out mice show defects in their cardiac and lymphatic vascular development and die post-natally (13, 14). CXCR4, with CXCL12 as its only chemokine ligand, is present on the cell membrane of most cell types. CXCL12 can therefore activate not only most leukocyte types, but also hematopoietic stem and progenitor cells, epithelial cells and endothelial cells (15–17). ACKR3 is an atypical receptor since it does not initiate signal transduction by the activation of G proteins, but by the recruitment of β-arrestins to the cell membrane (18). It binds CXCL12 and CXCL11 with high affinity and is expressed on hematopoietic cells, embryonic neuronal and cardiac tissues and activated endothelium (10, 19). Activation of ACKR3 leads to migration of neuronal progenitor cells and an increased cellular survival and adhesion (10, 20). Moreover, ACKR3 functions as a sink for CXCL12 and removes it from the environment, thus modifying chemokine gradients (21). To form these gradients, CXCL12 binds to negatively charged glycosaminoglycan (GAG) polymers on cell surfaces and in the extracellular matrix (22).

Since CXCL12 and its receptors are present throughout the body, its activity in homeostasis and inflammation needs to be thoroughly controlled. Several post-translational modifications have been described to reduce or completely abolish CXCL12 activity, including enzymatic NH_2_- and COOH-terminal truncation, Arg citrullination by peptidyl arginine deiminase (PAD) and Tyr nitration (23–26). Removal of NH_2_-terminal amino acids occurs by dipeptidyl peptidase-4 (DPP-4) or CD26, neutrophil elastase, cathepsin G and multiple members of the matrix metalloproteinase (MMP) family and inactivates CXCL12. COOH-terminal truncation occurs by the circulating carboxypeptidase N and the membrane-bound carboxypeptidase M, and reduces CXCL12 activity (23, 24). Most of these truncated CXCL12 forms have been detected in human plasma (27). The administration of the CXCR4 inhibitor AMD3100 together with granulocyte-colony stimulating factor (G-CSF) is a commonly used method to release progenitor cells from the bone marrow into the circulation. AMD3100 interrupts the interaction of CXCL12 with CXCR4, and CXCL12 is inactivated due to the induction of CD26 and neutrophil elastase by G-CSF (28, 29). *In vitro* citrullination of CXCL12 by incubation with PAD occurred rapidly and resulted in a reduced or abolished activity, depending on the number of citrullinated arginine residues. Co-localization of CXCL12 and PAD in biopsies of Crohn's disease patients suggests that this modification occurs *in vivo* (25).

Recently, nitration of both Tyr residues of CXCL12 by peroxynitrite was described (30). We noticed that CXCL12 may be degraded further by incubation with peroxynitrite. Additionally, we detected natural nitration of Tyr7 in the supernatant of co-cultures of CXCL12-producing bone marrow stromal cells and leukocytes under inflammatory conditions (26). Nitration of Tyr7 resulted in reduced calcium signaling, *in vitro* lymphocyte and monocyte chemotaxis and *in vivo* lymphocyte recruitment (26). In this study, we aimed to detect the nitrated amino acids of intact, non-degraded CXCL12 after incubation with peroxynitrite. CXCL12 variants with a nitrated Tyr61 were identified, synthesized and functionally evaluated *in vitro* and *in vivo*. Since CXCR4 is a main co-receptor for HIV-1 infection and CXCL12 functions as a natural competitor for HIV-1, we also tested the effects of nitration on the anti-HIV-1 capacity of CXCL12 (9). Finally, we evaluated the different nitrated CXCL12 variants in a chemotaxis assay with microvascular endothelial cells to reveal possible effects on angiogenesis.

## Materials and methods

### Cells

Chinese hamster ovary (CHO) cells, transfected with CXCR4 or ACKR3, were kindly provided by Prof. Dr. M. Parmentier from the Institute of Interdisciplinary Research in Human and Molecular Biology (IRIBHM) at the Université Libre de Bruxelles, Brussels, Belgium. CHO cells were cultivated in Ham's F-12 growth medium (Lonza, Basel, Switzerland) including 10% (v/v) FBS, 400 μg/ml G418 (Gibco, Auckland, New Zealand) and 250 μg/ml zeocin (Invitrogen, Carlsbad, CA). Human monocytic THP-1 cells (American Type Culture Collection, Manassas, VA, USA) were cultured in Roswell Park Memorial Institute medium 1640 with glutamine supplement (RPMI 1640–GlutaMAX; Gibco), which was further enriched with 10% (v/v) FBS and 3% (w/v) sodium bicarbonate. THP-1 and CHO cells were subcultivated 2 days prior to the signaling or cell migration assays. The C2C12 mouse myoblast cell line, stably transfected with CXCR4, was obtained from DiscoverX (Fremont, CA) and was cultured in Dulbecco's Modified Eagle's Medium 1885 (DMEM; Gibco), containing 20% (v/v) FBS, 1% (v/v) penicillin/streptomycin, 1% (v/v) L-glutamine, 500 μg/ml hygromycin B (Invitrogen) and 1,000 μg/ml G418 (Gibco). U2OS cells (ATCC) were cultured in Minimal Essential Medium alpha (MEM-alpha; Gibco) supplemented with 10% (v/v) FBS, 1% (v/v) L-glutamine, 1% (v/v) penicillin/streptomycin and 500 μg/ml hygromycin B. CV-1 in Origin with SV40 genes-7 (COS-7) cells (ATCC) were grown in DMEM 1885, supplemented with 10% (w/v) FBS, 1% (v/v) penicillin/streptomycin (Gibco) and 1% L-glutamine at 10% CO_2_ and 37°C. Human retinal microvascular endothelial cells (HRMVE; Cell Systems, Kirkland, WA, USA) were cultivated in endothelial basal medium-2 (EBM-2) with endothelial growth medium-2 MV (EGM2-MV) BulletKit supplement (Lonza). HRMVE cells were used at passage numbers between 5 and 8. Lymphocytes were isolated from buffy coats that were freshly provided by the Belgian Red Cross (Mechelen, Belgium) as previously described (31). Human lymphocytic MT-4 cells were cultured in RPMI 1640 (Gibco), supplemented with 10% (v/v) FBS. Unless stated otherwise, all cells were cultured at 5% CO_2_ and 37°C.

### Chemical synthesis of CXCL12 and nitrated variants

Native CXCL12 and its nitrated variants with a nitrotyrosine at position 7 or 61 or at both positions 7 and 61 were chemically synthesized using fluorenyl methoxycarbonyl (Fmoc) chemistry on an Activo-P11 solid phase peptide synthesizer (Activotec, Cambridge, UK) and subsequently deprotected, purified and folded as described previously (26).

### Chemical nitration of CXCL12

Synthetic CXCL12 was incubated with several concentrations of peroxynitrite (Cayman Chemical Company, Ann Arbor, MI, USA) at room temperature or 37°C for 1 min in a final volume of 30 μl ultrapure water. The reaction was stopped by adding 20 μl of 1% (v/v) trifluoroacetic acid (TFA; Biosolve, Valkenswaard, The Netherlands). Samples were injected from this reaction vial onto a Proto 300 C4 column (5 μm, 50 × 0.15 mm; Higgins Analytical, Mountain View, CA, USA), equilibrated with 0.1% (v/v) TFA and 4% (v/v) acetonitrile in ultrapure water. Elution occurred by reversed-phase ultra-high performance liquid chromatography (RP-UPLC; UltiMate 3000 RSLC; Thermo Scientific, Waltham, MA, USA), using a linear 0–80% acetonitrile gradient in 0.1% (v/v) formic acid. The location of nitrated Tyr in non-degraded CXCL12 was identified by detecting specific MS^2^ signature fragments in the eluate using an ion trap mass spectrometer (Amazon Speed ETD; Bruker Daltonics, Bremen, Germany).

### Glycosaminoglycan binding properties

The affinity of the different CXCL12 forms for heparin (Iduron, Macclesfield, UK) or chondroitin sulfate (Iduron) was measured with a previously described ELISA-like assay in GAG-binding plates (BD Biosciences, Franklin Lakes, NJ, USA) or by surface plasmon resonance (Biacore T200 instrument; GE Healthcare, Uppsala, Sweden) (32).

### CXCR4 binding and internalization

Binding of CXCL12 and its nitrated variants to CHO cells transfected with CXCR4 was investigated in a competition assay using CXCL12^AF647^ (Almac, Craigavon, Northern Ireland), as described previously (25). Internalization of CXCR4 after stimulation of CXCR4 transfected CHO cells or THP-1 cells with the CXCL12 variants was determined by detecting the remaining CXCR4 expression using specific phycoerythrin-labeled CXCR4 antibodies (Clone 12G5, BD Biosciences) or mouse anti human CXCR4 antibody clone 44717 (R&D Systems, Minneapolis, MN, USA) in combination with phycoerythrin-labeled goat anti mouse antibodies (BD Biosciences) and flow cytometry (FACSCalibur flow cytometer) (26).

### Signal transduction experiments

β-arrestin 2 recruitment to CXCR4 or ACKR3 after stimulation with the CXCL12 variants was tested in C2C12 cells, stably transfected with CXCR4, or U2OS cells, transiently transfected with ACKR3, respectively, using the β-galactosidase fragment complementation technology (PathHunter β-arrestin assay, DicoveRx) (26).

Calcium mobilization in THP-1 and CXCR4-transfected CHO cells was measured using the fluorescent calcium-binding dye Fura-2 (Molecular Probes, Invitrogen) on a LS50B spectrofluorimeter (PerkinElmer, Waltham, MA, USA) (33).

Phosphorylation of Akt and ERK1/2 was measured in cell lysates of CXCR4-transfected CHO cells stimulated at 37°C with the CXCL12 variants using specific phospho-Akt (S473) and phospho-ERK1(T202/Y204)/ERK2(T185/Y187) ELISA duosets (R&D Systems). The ratio of the concentration of phosphorylated kinase over the total amount of protein was calculated for each sample and depicted as percentage of unstimulated control.

The inositol turnover assay was performed with COS-7 cells, transiently transfected with CXCR4, by measuring the amount of radioactive inositol in the cytoplasm after stimulation of the cells with the CXCL12 variants, as previously described (26).

### Chemotaxis assays

Migration of THP-1 cells toward different CXCL12 variants was tested in 96-well multiscreen plates (Millipore, Billerica, MA, USA). Migrated cells were quantified as previously described using the ATPlite kit (PerkinElmer) (34). Chemotactic indices were calculated as the ratio of the luminescence intensity of cells migrated toward the test sample over the luminescence intensity of cells migrated toward chemotaxis buffer alone.

Lymphocyte chemotaxis in Boyden microchambers using fresh, unfractionated PBMCs was performed under conditions that favor lymphocyte migration, i.e., a 4 h incubation period that allowed 10 × 10^6^ PBMCs/ml to migrate through 5 μm pores of a PVP-free polycarbonate membrane coated with 25 μg/ml fibronectin (35). Migrated cells were fixed, colored with Hemacolor solutions (Merck, Darmstadt, Germany) and counted microscopically. Chemotactic indices were calculated as the ratio of cells migrated to chemokine solution and cells migrated to buffer solution.

Migration of HRMVE cells was evaluated using cell invasion-migration (CIM) plates and the real-time cell analyzer double plate xCELLigence instrument (RCTA-DP; xCELLigence System; Acea Biosciences, San Diego, CA, USA) (32). For each experiment, performed in duplicate, the cell indices after 15 h migration were normalized to the cell index of unstimulated endothelial cells (set at 100%).

### *In vivo* lymphocyte extravasation

While being anesthetized using 3.75% (w/v) ketamine (Syntec, Santano de Parnaíba, Brazil) and 0.25% (w/v) xylazine (Syntec) in PBS, endotoxin-free (detection limit: 0.125 pg LPS per μg chemokine) synthetic CXCL12 variants were injected into the knee cavity of 8 weeks old C57BL/6 mice (Animal Care Center of the Universidade Federal de Minas Gerais, Brazil). The mice received water containing 1.7 mg/ml of the CD26 inhibitor sitagliptin [Merck Sharpe & Dohme (MSD), Whitehouse Station, NJ, USA] during 3 days prior to injection. Three hours after the injection, the mice were sacrificed by a subcutaneous injection of a ketamine/xylazine overdose. The cells that migrated to the tibiofemoral articulation were harvested and counted differentially (26). The *in vivo* protocols using laboratory animals were reviewed and approved by the Animal Ethical Committee of the University of Minas Gerais and Belgian, European and Brazilian guidelines regarding laboratory animal handling were followed.

### Enzyme incubations

CXCL12 and its nitrated variants, at a final concentration of 5 μM, were incubated for several time intervals with CD26, carboxypeptidase M (CPM; R&D Systems) and matrix metalloproteinase-9 (MMP-9) at final concentrations of, respectively, 1.65 nM (5 U/L), 15 nM and 10 nM. The incubations with CD26 and CPM were performed in a buffer containing 50 mM Tris/HCl and 1 mM EDTA at pH 7.5. A buffer containing 150 mM NaCl, 50 mM Tris and 10 mM CaCl_2_ (pH 7.5) was used for incubations with MMP-9. All incubations were performed at 37°C. The reaction was stopped by adding 20 μl 0.1% (v/v) TFA. Hereafter, samples were desalted using C18 ZipTips (Millipore) and eluted with 50% (v/v) acetonitrile in 0.1% (v/v) formic acid. The samples were injected in an Amazon Speed ETD mass spectrometer (Bruker Daltonics) and analyzed using Bruker deconvolution software. An abundance cut-off of 5% and a minimum of four peaks per protein was maintained. Intensities of the deconvoluted peaks were used to quantify the chemokine after incubation.

### Anti-HIV assays

The inhibitory effect of the CXCL12 variants on the infection of MT-4 cells with the CXCR4-using HIV-1 strain NL4.3 was assessed. 5-fold dilutions of the CXCL12 variants were applied to a flat-bottom 96-well plate, after which 7.5 × 10^4^ MT-4 cells were added in 50 μl medium. After adding 50 μl of diluted HIV-1 stock (500 pg/ml p24 Ag), cells were incubated for 4 days until a strong cytopathic effect was observed in the untreated HIV-1-infected cells. Antiviral activity of the CXCL12 variants was measured by the MTS method (36). IC_50_ values of the CXCL12 variants were calculated.

### Statistical analyses

All statistical analyses to determine differences between CXCL12 variants were performed using the Mann-Whitney *U* test.

## Results

### Nitration of tyr7 and tyr61 of CXCL12 was detected after incubation with peroxynitrite

**Figure 1:**
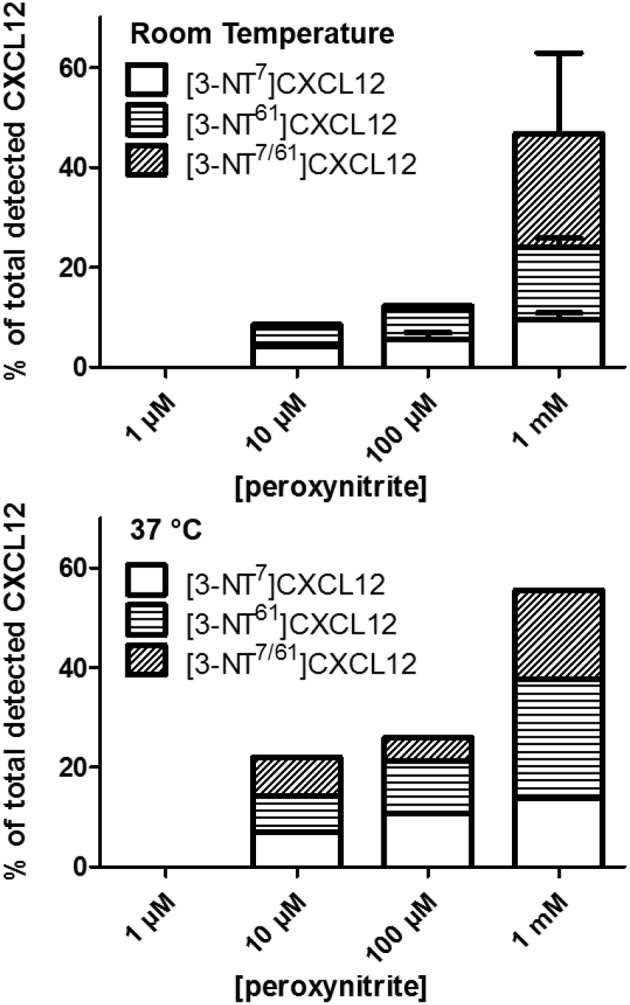
Detection of nitrated CXCL12 forms by MS^2^, following peroxynitrite incubation. CXCL12 was incubated with concentrations ranging from 1 μM to 1 mM peroxynitrite for 1 min at room temperature (upper panel) or 37°C (lower panel). A sample of the reaction volume was analyzed using RP-HPLC coupled to an ion trap mass spectrometer. The presence of CXCL12, [3-NT^7^]CXCL12, [3-NT^61^]CXCL12 and [3-NT^7/61^]CXCL12 was determined by measuring variant-specific peptide fragments after MS^2^ fragmentation of the intact protein. The amount of each nitrated CXCL12 form is shown as the percentage of the sum of all detected CXCL12 forms.

Since peroxynitrite can nitrate any amino acid with a ring structure, we investigated whether additionally to the reported Tyr7, nitrated Trp57 and/or Tyr61 could be detected without complete degradation of intact protein. SDS-PAGE quality control showed no protein degradation after a 1 min incubation with any of the used peroxynitrite concentrations. Nitration of Tyr or Trp results in the addition of 45 Da to the protein, whereas the charge remains the same. By mass spectrometry, we detected that the molecular mass of CXCL12 was unaltered or increased with 45 or 90 mass units, corresponding with none, one or two nitrated residues. Proteins were fragmented using collision-induced dissociation in the mass spectrometer in such a way that only the Asp-Pro bond in the protein was cleaved. This resulted in a NH_2_-terminal MS^2^ fragment containing Tyr7 and a COOH-terminal MS^2^ fragment containing Trp57 and Tyr61. The analysis of the mass of these fragments allowed to determine whether they carried a nitrated amino acid and MS^3^ analysis enabled us to locate the position of nitration in the COOH-terminal fragment, i.e., Trp57 or Tyr61. Using multiple reaction monitoring (MRM), the eluate of the peroxynitrite-incubated sample that was loaded on the HPLC was scanned by automated MS^2^ for CXCL12 variants with none, one, two or three additional nitro compounds. The results of the incubation of CXCL12 with various peroxynitrite concentrations at room temperature are shown in Figure 1. An incubation with 1 μM peroxynitrite did not nitrate CXCL12, whereas the amount of single nitrated, [3-NT^7^]CXCL12 and [3-NT^61^]CXCL12, and double nitrated CXCL12, [3-NT^7/61^]CXCL12, increased gradually with peroxynitrite concentrations increasing from 10 μM to 1 mM. No CXCL12 with three nitrated amino acids was detected. MS^3^ analysis on the COOH-terminal MS^2^ fragment containing one nitro compound confirmed that Tyr61, and not Trp57, was nitrated. No COOH-terminal CXCL12 MS^2^ fragment with two nitrated amino acids (90 mass units extra) was detected. The same effect was observed by incubating CXCL12 with these peroxynitrite concentrations at 37°C (Figure 1). However, compared to room temperature more nitrated CXCL12 was generated in the same period of time when using 10 or 100 μM peroxynitrite. The total amount of nitrated CXCL12 after incubation with 1 mM peroxynitrite at 37°C was 55%, similar to the detected amount of nitrated CXCL12 after incubation with 1 mM at room temperature.

### Production of synthetic CXCL12 variants with nitration on tyr61

**Figure 2:**
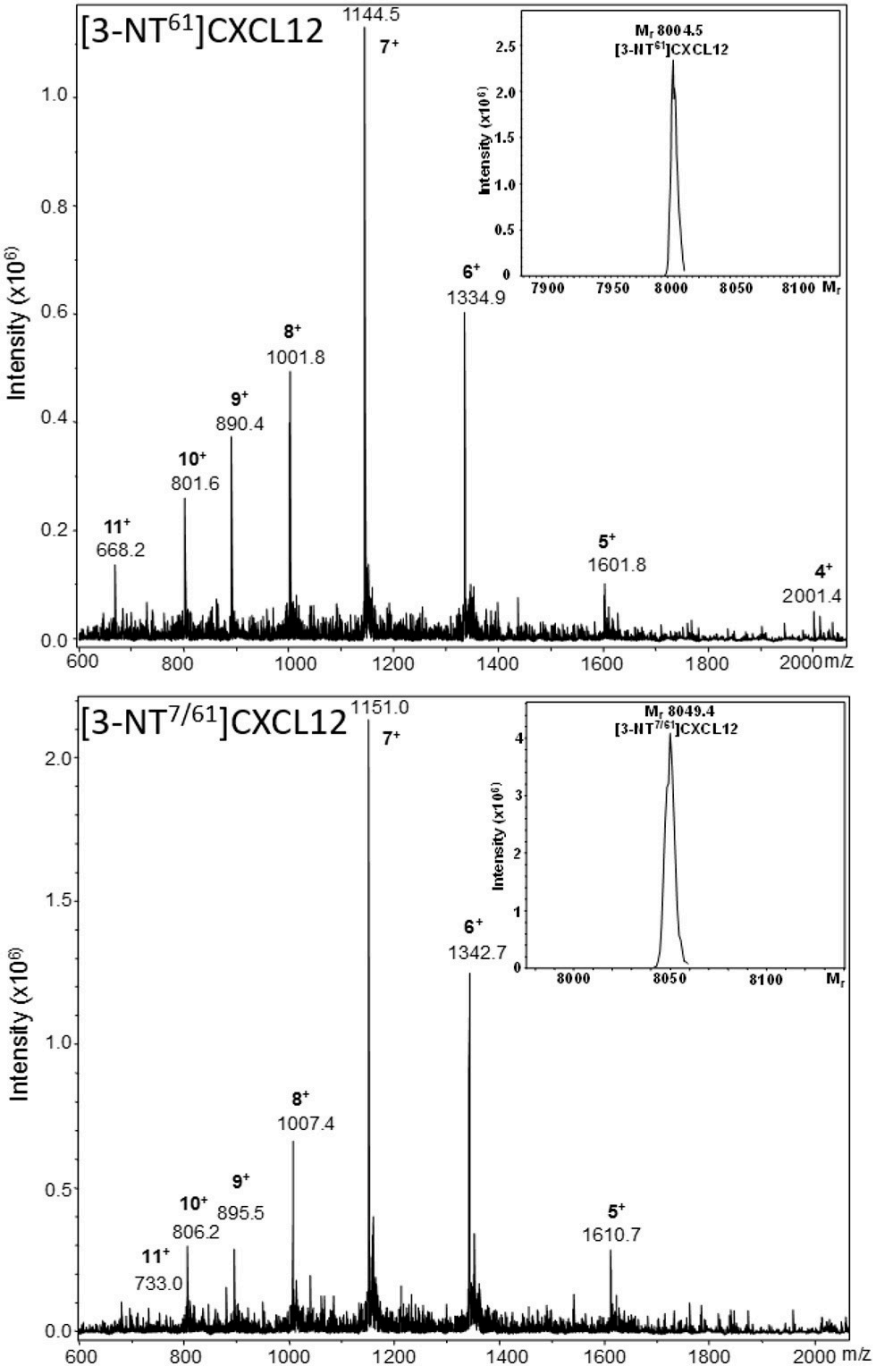
Mass spectrometric analysis of synthetic [3-NT^61^]CXCL12 and [3-NT^7/61^]CXCL12. [3-NT^61^]CXCL12 and [3-NT^7/61^]CXCL12 were synthesized, deprotected, folded and purified using RP-HPLC. Fractions that contained pure protein with the correct relative molecular mass (M_r_) were selected and pooled. The averaged mass spectrum of the final pool of folded and purified [3-NT^61^]CXCL12 and [3-NT^7/61^]CXCL12 are shown in the upper and lower panel, respectively. The number of charges and mass to charge ratios (m/z) of the detected ions are indicated, together with the ion intensities. The inserts in both panels show the deconvoluted mass spectra with the M_r_ of the uncharged proteins, as determined using Bruker deconvolution software.

Two new CXCL12 variants containing nitrated Tyr61 were synthesized using Fmoc solid phase peptide synthesis: one form with only Tyr61 nitrated, i.e., [3-NT^61^]CXCL12, and another form with both Tyr7 and Tyr61 nitrated, i.e., [3-NT^7/61^]CXCL12. The synthetic proteins were folded and purified by reversed-phase (RP)-HPLC. The M_r_ of each of these forms was confirmed by ion trap mass spectrometry (Figure 2). [3-NT^61^]CXCL12 and [3-NT^7/61^]CXCL12 had an experimental mass of 8,004.5 and 8,049.4, respectively (theoretical M_r_ 8,004.4 and 8,049.4, respectively). Native CXCL12 and CXCL12 nitrated at Tyr7, [3-NT^7^]CXCL12 were produced before (26).

### Nitrated CXCL12 has higher affinity for chondroitin sulfate compared to native CXCL12

**Figure 3:**
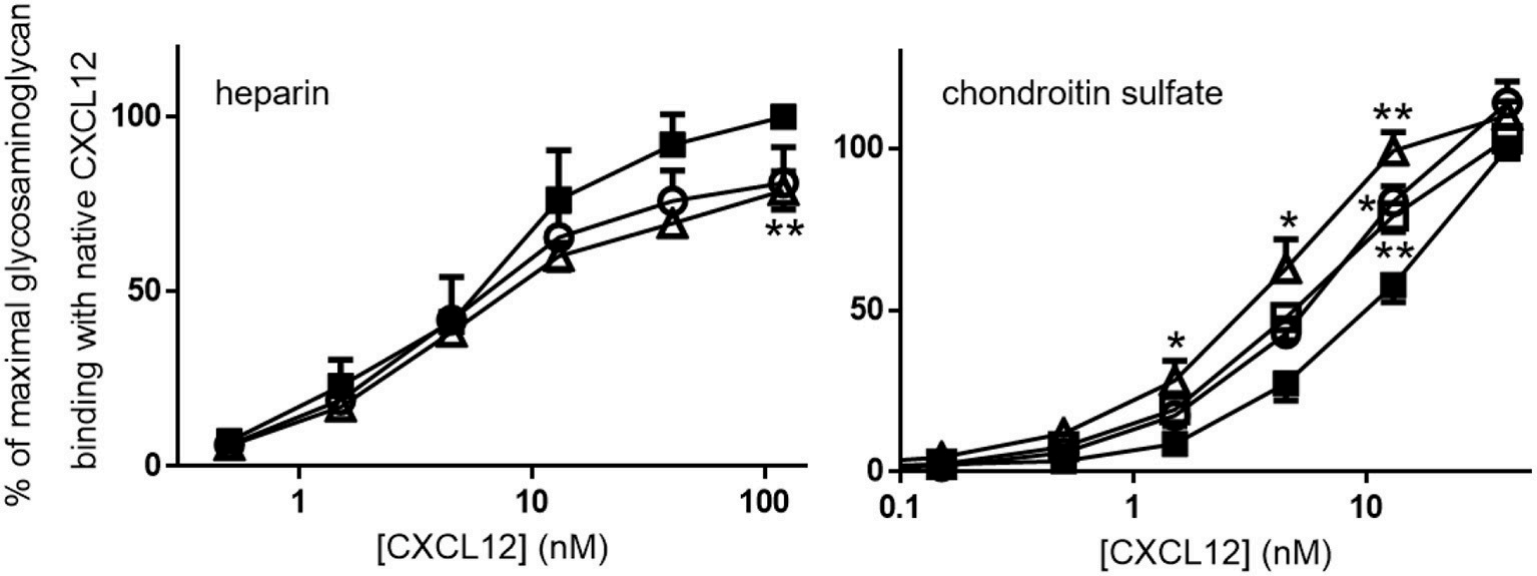
CXCL12 nitration enhances binding to plates coated with chondroitin sulfate, but not with heparin, Serial dilutions of CXCL12 (■, filled squares), [3-NT^7^]CXCL12 (□, open squares), [3-NT^61^]CXCL12 (°, open circles) and [3-NT^7/61^]CXCL12 (Δ, open triangles) were incubated in heparin-coated or chondroitin sulfate-coated plates. Bound CXCL12 forms were detected using biotinylated anti-human CXCL12 and horse radish peroxidase-labeled streptavidin and represented as the mean percentage (*n* = 3 for heparin, *n* = 7 for chondroitin sulfate, ±SEM) of the absorbance of 120 nM (for the heparin experiments) or 40 nM (for the chondroitin sulfate experiments) CXCL12 (at λ = 450 nm). Statistically significant differences between the nitrated CXCL12 forms and native CXCL12 were determined using the Mann-Whitney *U* test (*p < 0.05, **p < 0.01).

**Figure 4:**
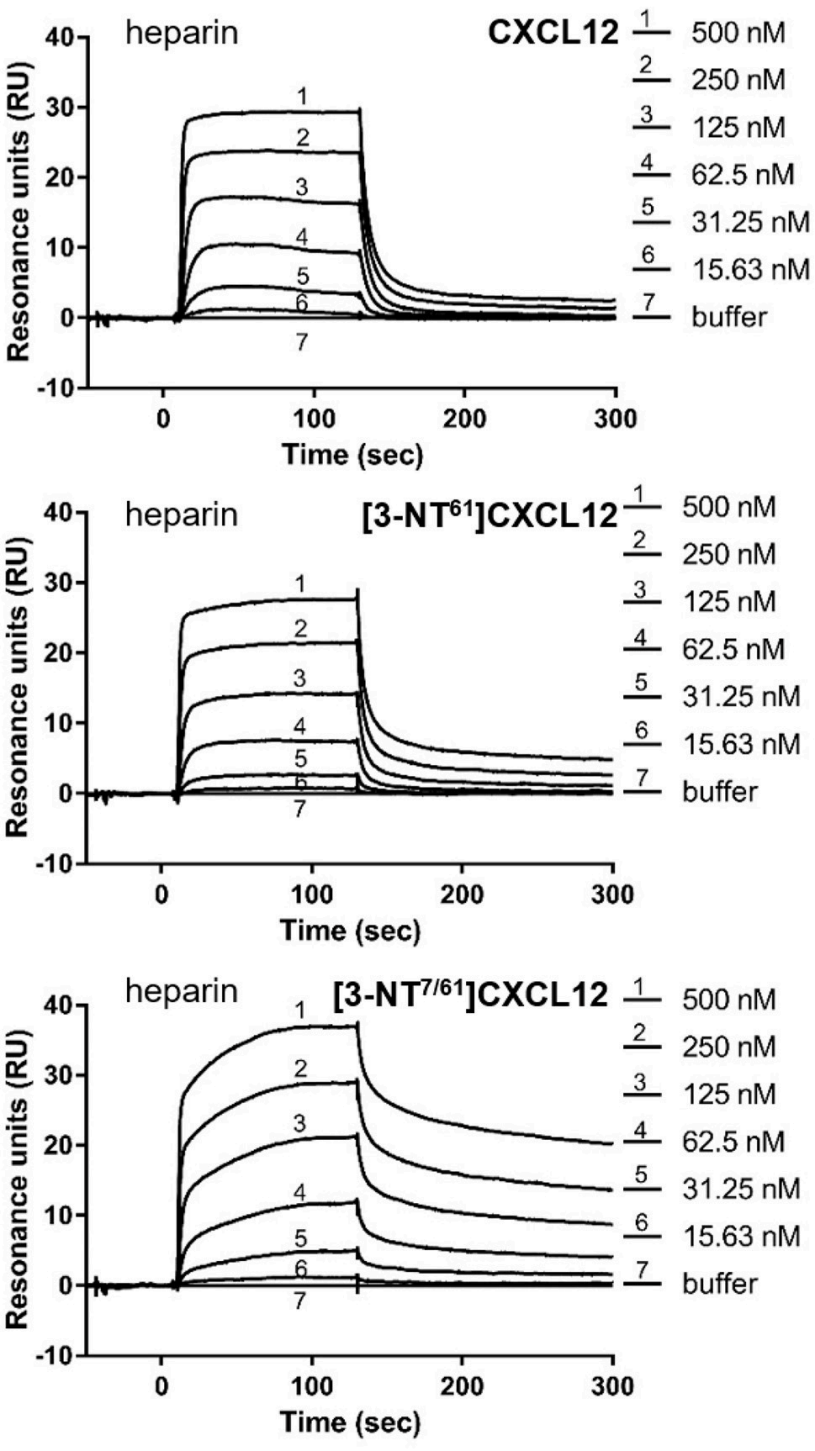
Surface plasmon resonance reveals comparable binding to heparin for unmodified and nitrated CXCL12. Surface plasmon resonance (SPR) analysis of the interaction of several concentrations (15.63–500 nM) of CXCL12, [3-NT^61^]CXCL12 and [3-NT^7/61^]CXCL12 with heparin-coated sensor chips are shown. One representative experiment out of two is displayed.

The binding affinities of [3-NT^61^]CXCL12 and [3-NT^7/61^]CXCL12 for heparin and the binding affinities of [3-NT^7^]CXCL12, [3-NT^61^]CXCL12, and [3-NT^7/61^]CXCL12 for chondroitin sulfate were compared to CXCL12. Whereas, there was a tendency toward lower heparin binding for high concentrations of [3-NT^61^]CXCL12 and [3-NT^7/61^]CXCL12, there was only a significant difference in heparin binding between 120 nM of CXCL12 and [3-NT^7/61^]CXCL12. Chondroitin sulfate binding, however, was significantly higher at 13 nM for [3-NT^7^]CXCL12 and CXCL12 [3-NT^61^]CXCL12 and at 1.5, 4.5, and 13 nM for [3-NT^7/61^]CXCL12 compared to the same concentrations of CXCL12 (Figure 3). Steady state analysis of the SPR binding kinetics on heparin (Figure 4) also showed similar K_D_ values for all CXCL12 forms. Whereas, the K_D_ calculated for CXCL12 on heparin was 125 nM, the K_D_ for [3-NT^61^]CXCL12 and [3-NT^7/61^]CXCL12 was 157 nM and 133 nM, respectively.

**Figure 5:**
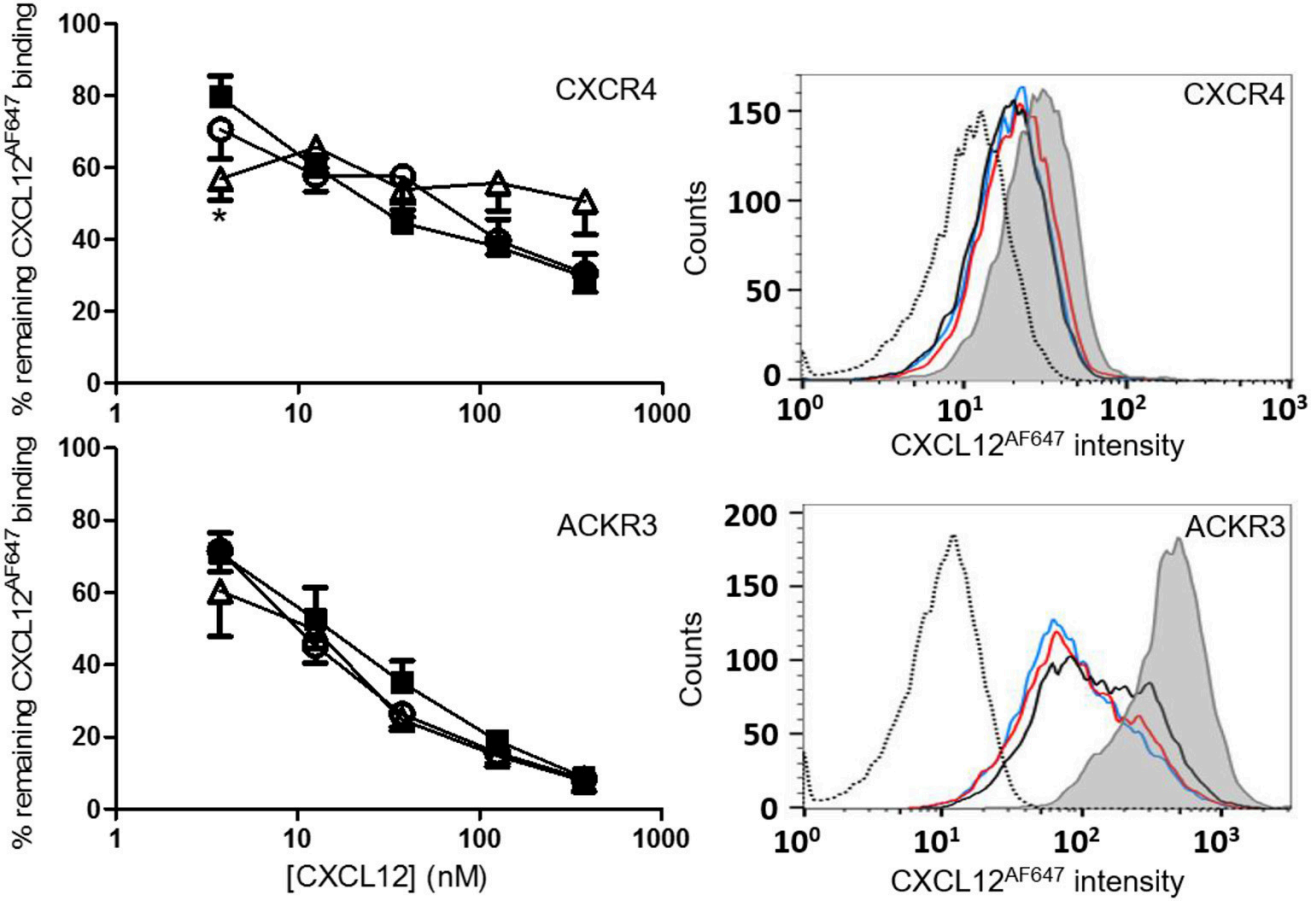
Binding of CXCL12 forms to CXCR4 and ACKR3. Binding of CXCL12 (■, filled squares), [3-NT^61^]CXCL12 (°, open circles) and [3-NT^7/61^]CXCL12 (Δ, open triangles) to CXCR4 was tested by adding various concentrations (ranging from 3.75 to 375 nM) of these CXCL12 forms together with 12.5 nM CXCL12^AF647^ to CHO cells transfected with CXCR4. Binding is expressed as average fluorescent intensity relative to maximal fluorescence, obtained by incubation of the cells with 12.5 nM CXCL12^AF647^ without competitor. Mean percentages (±SEM) are derived from eight independent experiments. Flow cytometry data from a representative receptor binding experiment show the remaining fluorescence of CXCL12^AF647^ after competition with 37.5 nM CXCL12 (black line), 37.5 nM [3-NT^61^]CXCL12 (red line) or 37.5 nM [3-NT^7/61^]CXCL12 (blue line). The histogram where only CXCL12^AF647^ was added to the cells (gray area) shows the maximal fluorescence. The unstained control is shown as a dotted histogram. Similar binding experiments were performed using CHO cells transfected with ACKR3 (lower panels). Various concentrations of CXCL12 (■, filled squares), [3-NT^61^]CXCL12 (°, open circles) and [3-NT^7/61^]CXCL12 (Δ, open triangles) were added together with 12.5 nM CXCL12^AF647^ to compete for ACKR3 binding. Binding is expressed relatively to the maximal fluorescence of CXCL12^AF647^ alone (*n* = 4, ±SEM). Flow cytometry data from a representative experiment show the remaining CXCL12^AF647^ fluorescence after competition with 37.5 nM of the various CXCL12 variants [color coding as indicated in the upper panel]. Statistically significant differences between the nitrated CXCL12 forms and native CXCL12 were determined using the Mann-Whitney *U* test (*p < 0.05).

Binding of these nitrated CXCL12 forms to their 7TM receptors CXCR4 and ACKR3 was tested in CXCR4-transfected and ACKR3-transfected CHO cells (Figure 5). Competition of CXCL12 and [3-NT^61^]CXCL12 with 12.5 nM of fluorescently labeled CXCL12^AF647^ was comparable for both receptors. The ability to compete with CXCL12^AF647^ for CXCR4 binding was slightly reduced for [3-NT^7/61^]CXCL12 compared to CXCL12, whereas no significant differences were detected between these forms regarding ACKR3 binding. This comparable binding is demonstrated by a representative flow cytometry experiment showing raw data for each of the receptors (Figure 5).

### β-arrestin 2 recruitment to CXCR4, but not to ACKR3, is reduced for [3-NT^7/61^]CXCL12

**Figure 6:**
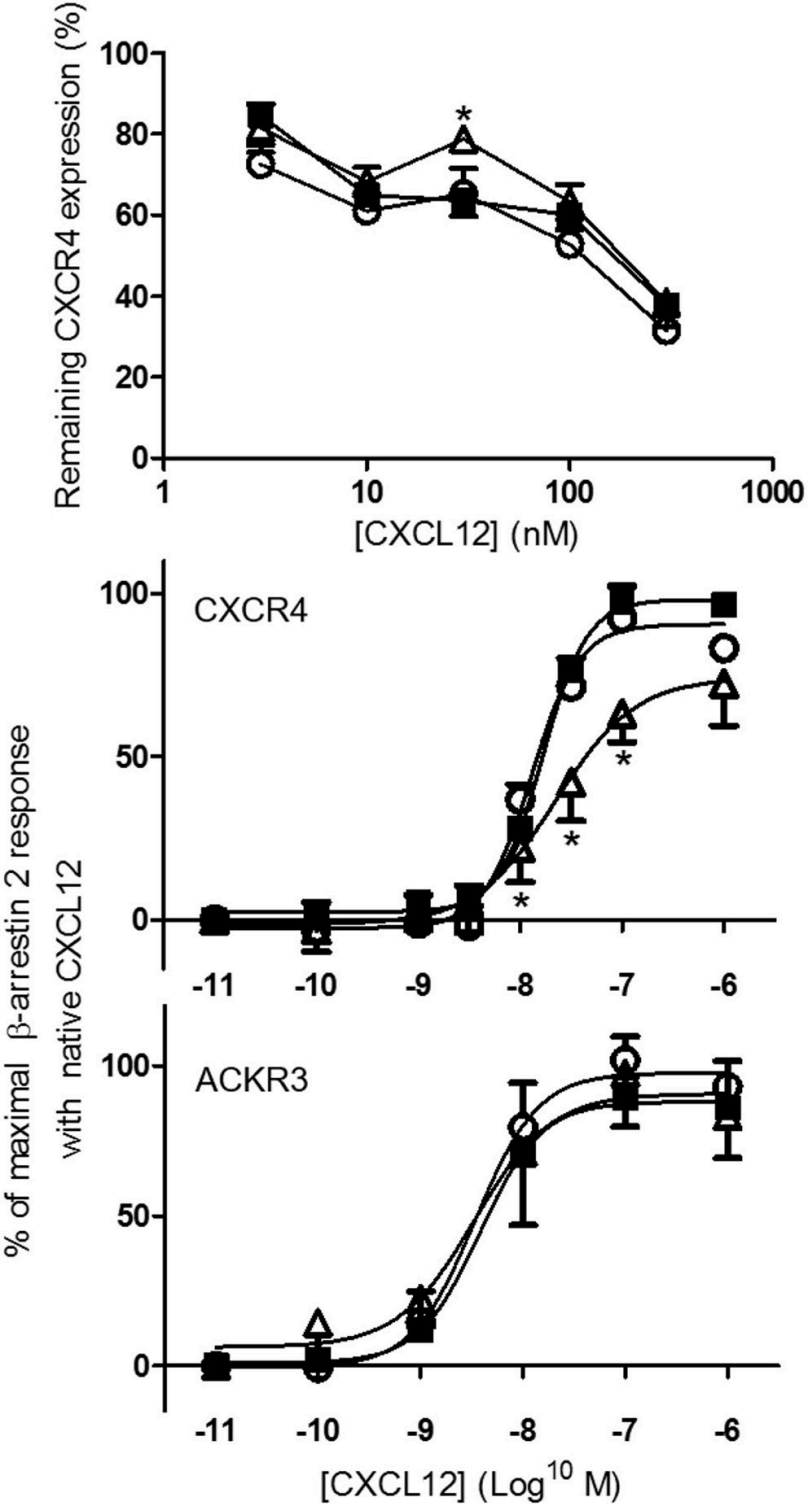
Internalization of CXCR4 and recruitment of β-arrestin 2 to CXCR4 and ACKR3 following stimulation with native or nitrated CXCL12. CXCL12 (■, filled squares), [3-NT^61^]CXCL12 (°, open circles) and [3-NT^7/61^]CXCL12 (Δ, open triangles) were compared for their potency to induce CXCR4 internalization (upper panel) and to recruit β-arrestin 2 to CXCR4 and ACKR3 (lower panels). CHO-CXCR4 cells were incubated for 1 h at 37°C with various concentrations (from 3.75 to 375 nM) of the different CXCL12 forms (upper panel). The remaining receptor expression of CXCR4 was measured using specific CXCR4 antibodies (clone 12G5) and shown as an average fluorescent intensity relative to the maximal fluorescence, obtained by measuring CXCR4 expression without ligand stimulation (*n* = 4, ±SEM). The recruitment of β-arrestin 2 was measured in CXCR4-transfected C2C12 cells (*n* ≥ 4) and ACKR3-transfected U2OS cells (*n* = 3) after stimulation with 10-fold serial dilutions (0.01 nM−1 μM) of the CXCL12 forms (lower panels). The mean percentage (±SEM) of the maximal β-arrestin 2 recruitment with native CXCL12 is shown. Statistical differences between native CXCL12 and the nitrated CXCL12 forms were determined using the Mann-Whitney *U* test (*p < 0.05).

In addition to receptor binding, the ability of CXCL12, [3-NT^61^]CXCL12, and [3-NT^7/61^]CXCL12 to induce internalization of CXCR4 was investigated using antibody clones 12G5 and 44717 and compared. The dose-dependent capacity of the three CXCL12 forms to induce CXCR4 internalization on CXCR4 transfected CHO cells detected with anti CXCR4 clone 12G5 is shown in Figure 6 (upper panel). A comparable receptor internalization was detected with antibody clone 44717 upon treatment of CHO CXCR4 cells with 375 nM CXCL12 than with clone 12G5 (Figure 6 upper panel and data not shown). Only at 37.5 nM [3-NT^7/61^]CXCL12 was significantly weaker at inducing CXCR4 internalization than native CXCL12. Up to 375 nM CXCL12 failed to induce significant CXCR4 internalization on THP-1 cells (data not shown). Since receptor internalization is mediated by recruiting β-arrestin to the cell membrane and [3-NT^7^]CXCL12 recruited β-arrestin 2 less efficiently than CXCL12 (26), we tested how [3-NT^61^]CXCL12 and [3-NT^7/61^]CXCL12 compared to CXCL12 regarding β-arrestin 2 recruitment (Figure 6 middle and lower panel). Whereas, no difference in β-arrestin 2 recruitment to ACKR3 was measured among these CXCL12 forms, [3-NT^7/61^]CXCL12 recruited significantly less β-arrestin 2 to CXCR4 compared to native CXCL12.

### [3-NT^7/61^]CXCL12 shows a reduced calcium signaling potency compared to native CXCL12

**Figure 7:**
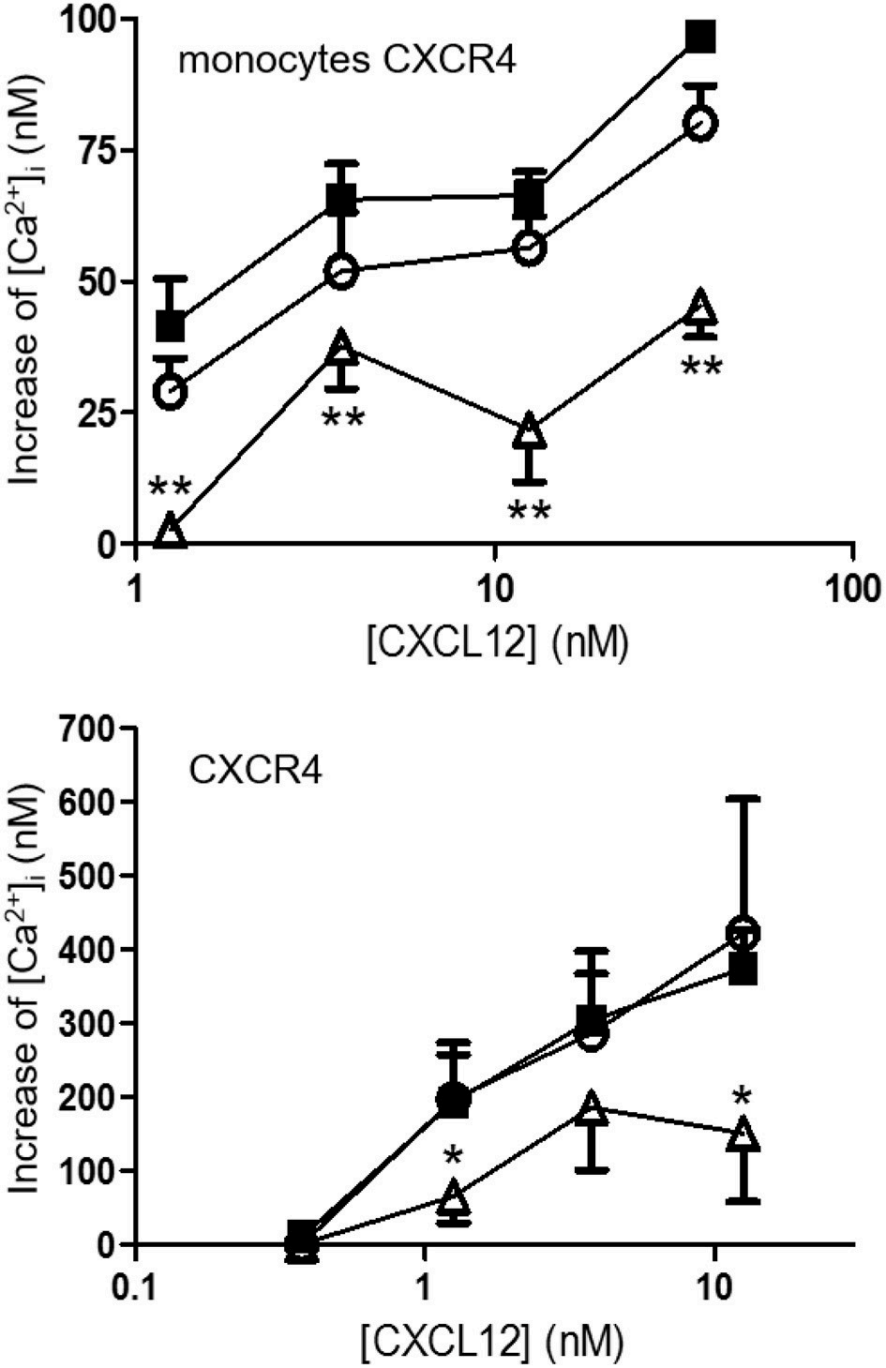
Calcium mobilization following CXCR4 activation with different forms of CXCL12. The increase of the intracellular calcium concentration [Ca^2+^]_i_ after stimulation of monocytic THP-1 cells (upper panel; *n* = 6; ±SEM) and CXCR4-transfected CHO cells (lower panel; *n* = 7, ±SEM) with concentrations ranging from 0.375 to 37.5 nM of CXCL12 (■, filled squares), [3-NT^61^]CXCL12 (°, open circles) or [3-NT^7/61^]CXCL12 (Δ, open triangles) was measured. Statistical analyses, comparing [3-NT^61^]CXCL12 and [3-NT^7/61^]CXCL12 to CXCL12, were performed using the Mann-Whitney *U* test (*p < 0.05, **p < 0.01).

CXCL12, [3-NT^61^]CXCL12 and [3-NT^7/61^]CXCL12 were compared in several signaling pathways. The increase of the intracellular calcium concentration ([Ca^2+^]_i_) following stimulation of CXCR4 was measured in monocytic THP-1 cells and CXCR4-transfected CHO cells. In THP-1 cells, every dose of CXCL12 induced a significantly higher increase of [Ca^2+^]_i_ compared to [3-NT^7/61^]CXCL12. CXCL12 and [3-NT^61^]CXCL12 were equally potent Ca^2+^ mobilizers (Figure 7 upper panel). Also on CHO-CXCR4 cells, [3-NT^7/61^]CXCL12 was significantly less potent to increase the [Ca^2+^]_i_ compared to CXCL12, whereas also on these cells [3-NT^61^]CXCL12 had a similar potency as CXCL12 (Figure 7 lower panel).

**Figure 8:**
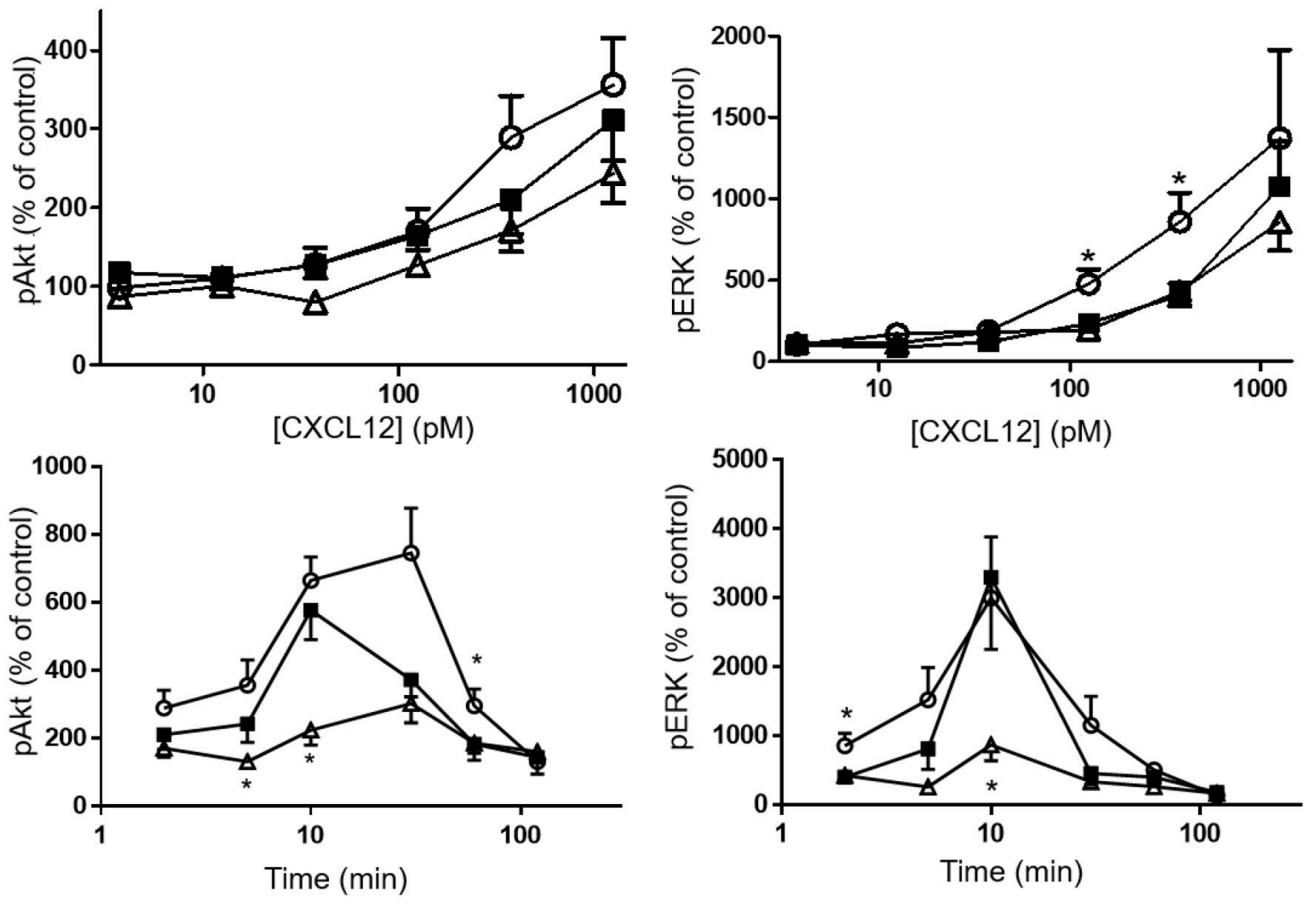
Effect of CXCL12 nitration on phosphorylation of Akt and ERK following CXCR4 activation. The amount of phosphorylated second messengers Akt (left panels) and ERK1/2 (right panels) was measured after a 2 min stimulation (upper panels) of CXCR4-transfected CHO cells with CXCL12 (■, filled squares), [3-NT^61^]CXCL12 (°, open circles) or [3-NT^7/61^]CXCL12 (Δ, open triangles) at concentrations between 3.75 pM and 1.25 nM. Alternatively, CXCR4-transfected CHO cells were stimulated for 2–120 min with 375 pM of the above mentioned CXCL12 forms (lower panels). The average accumulation (*n* = 6–8, ±SEM) of phosphorylated Akt and ERK1/2 was calculated as a percentage of vehicle-stimulated control. Statistical analyses, comparing [3-NT^61^]CXCL12 and [3-NT^7/61^]CXCL12 to CXCL12, were performed using the Mann-Whitney *U* test (*p < 0.05).

Phosphorylation of the kinases Akt and ERK1/2 was tested after stimulating CHO-CXCR4 cells for 2 min with various concentrations of CXCL12, [3-NT^61^]CXCL12 and [3-NT^7/61^]CXCL12 (Figure 8 upper panels). No significant differences in Akt phosphorylation were measured when cells were stimulated for 2 min with these CXCL12 forms. Surprisingly, stimulation of CXCR4 for 2 min with 125 and 375 pM [3-NT^61^]CXCL12 caused a significantly higher ERK1/2 phosphorylation compared to the same doses of CXCL12. Similar potencies in inducing phosphorylation of ERK1/2 were detected for CXCL12 and [3-NT^7/61^]CXCL12 after 2 min. Longer incubation of the cells resulted in more pronounced differences between the three CXCL12 forms (Figure 8 lower panels). The trend toward higher activity for [3-NT^61^]CXCL12 in Akt and ERK phosphorylation assays after 2 min was confirmed at later time points. In addition, [3-NT^7/61^]CXCL12 was clearly less potent than unmodified CXCL12 after 10 min in both signaling assays.

**Figure 9:**
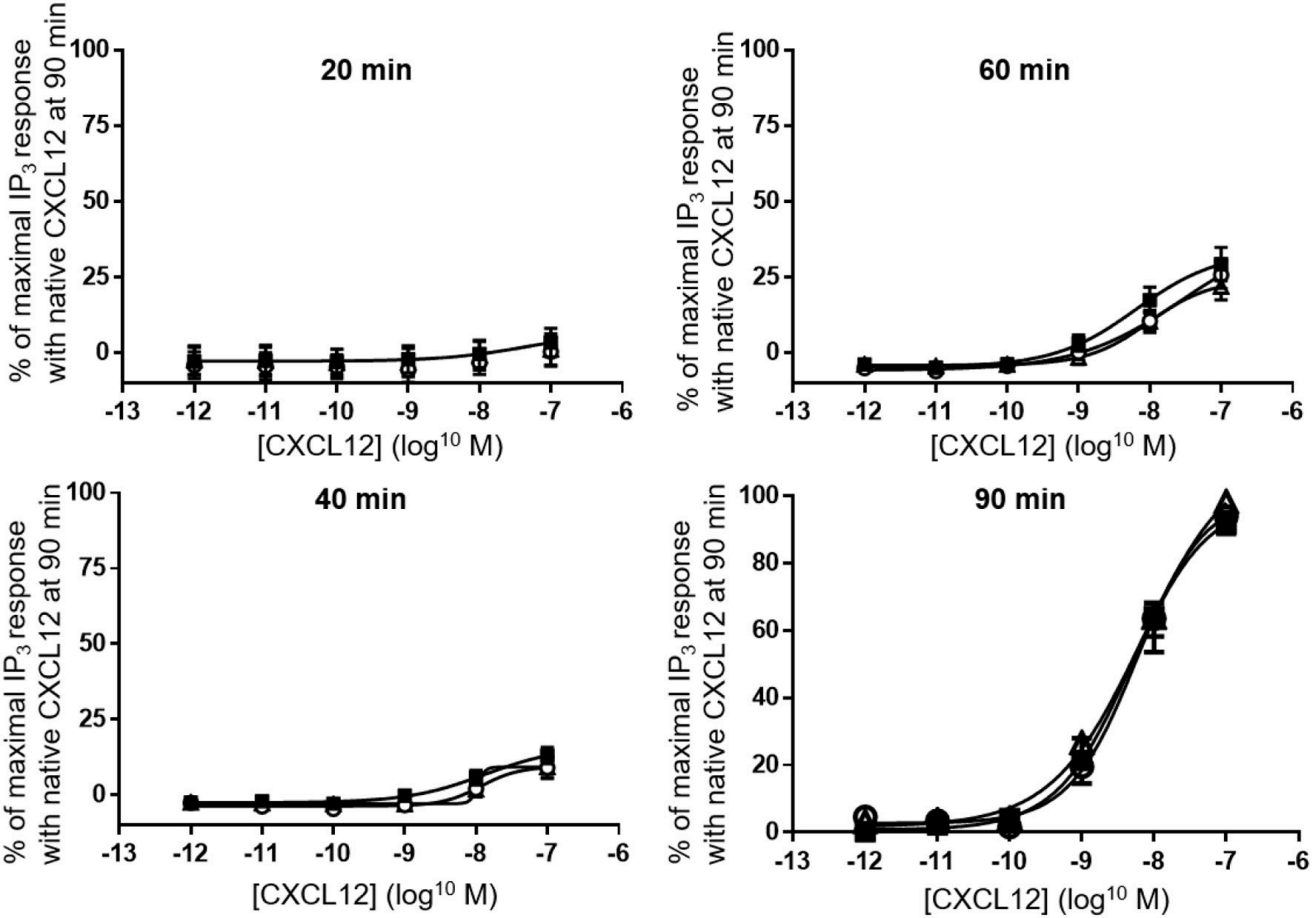
Accumulation of IP_3_ following CXCR4 activation with different forms of CXCL12. Accumulation of IP_3_ was measured in the cytoplasm after 20, 40, 60 (all *n* = 2), or 90 (*n* = 6, ±SEM) min stimulation of CXCR4 with serial dilutions of CXCL12 (■, filled squares), [3-NT^61^]CXCL12 (°, open circles) or [3-NT^7/61^]CXCL12 (Δ, open triangles).

Finally, despite the clear differences in calcium signaling that we observed in both THP-1 cells and CHO-CXCR4 cells, stimulation of CXCR4-transfected COS-7 cells for 20, 40, 60 or 90 min showed an equal ability of all CXCL12 forms to generate and accumulate IP_3_ in the cytoplasm (Figure 9).

### [3-NT^7/61^]CXCL12 is less chemotactic for monocytic THP-1 cells, lymphocytes and endothelial cells compared to CXCL12

**Figure 10:**
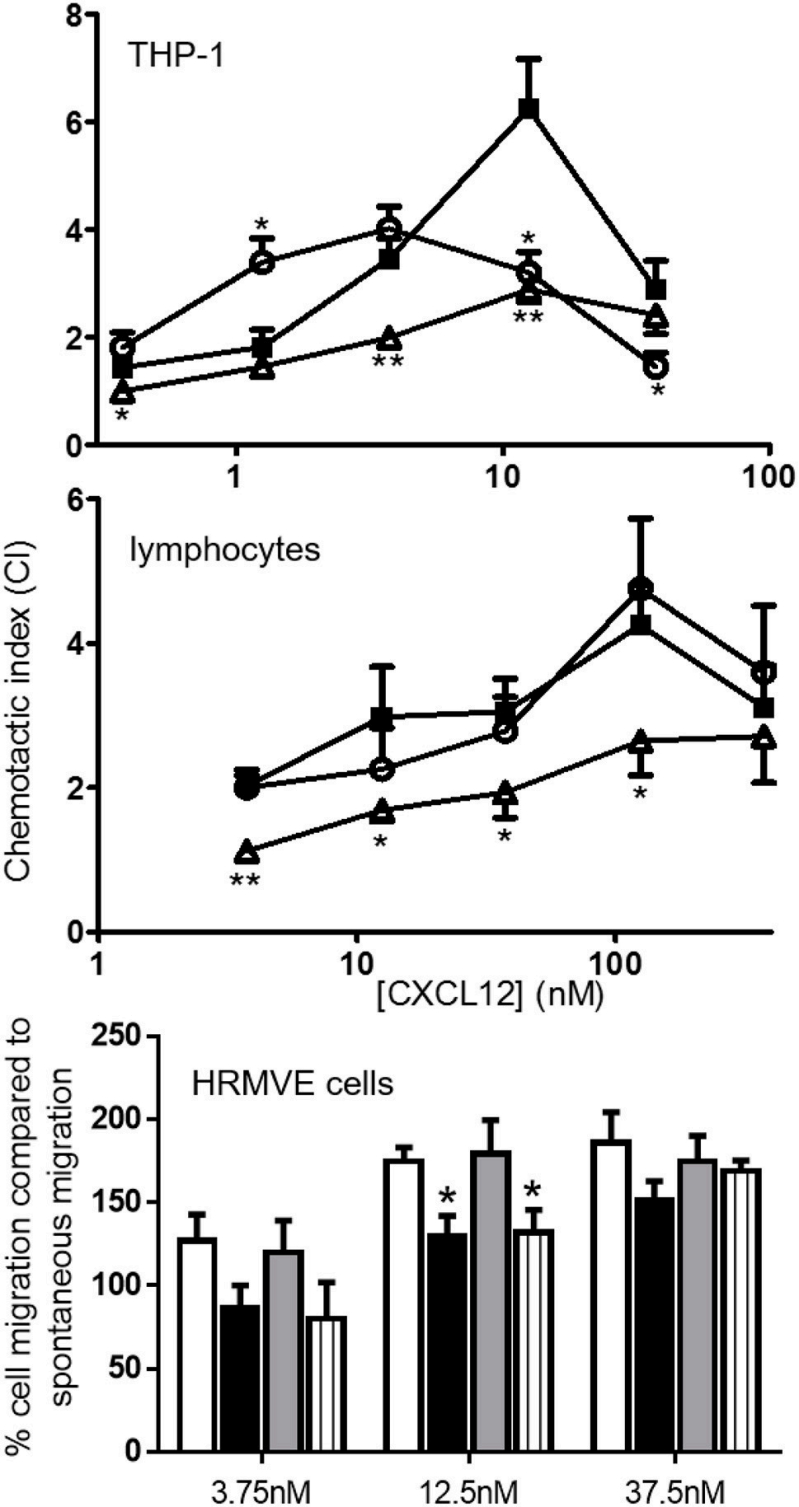
*In vitro* monocytic, lymphocyte and endothelial cell chemotaxis toward CXCL12 and its nitrated variants. CXCL12 (■, filled squares), [3-NT^61^]CXCL12 (°, open circles) and [3-NT^7/61^]CXCL12 (Δ, open triangles) were compared for their ability to induce chemotaxis of monocytic THP-1 cells (top panel; *n* ≥ 7) and freshly isolated lymphocytes (middle panel, *n* ≥ 7). The data are represented by chemotactic indices (mean ± SEM). *In vitro* migration of endothelial cells (lower panel) was investigated by measuring the changes in electrical impedance caused by cellular migration after stimulation of the cells with CXCL12 (white bars), [3-NT^7^]CXCL12 (black bars), [3-NT^61^]CXCL12 (gray bars) and [3-NT^61^]CXCL12 (striped bars). The data are represented as a mean percentage (*n* ≥ 4, ±SEM) of cell migration compared to spontaneous migration. Statistical comparison between CXCL12 and its nitrated forms was performed with the Mann-Whitney *U* test (*p < 0.05, **p < 0.01).

As a result of receptor activation, chemokines usually induce chemotaxis of their target cells. Therefore, the chemotactic potency of [3-NT^61^]CXCL12 and [3-NT^7/61^]CXCL12 for monocytic THP-1 cells, lymphocytes and endothelial cells was compared with that of CXCL12. Chemotaxis assays using THP-1 cells showed that both CXCL12 and [3-NT^61^]CXCL12 were able to induce a significant monocyte chemotactic response at any given dose (Figure 10 upper panel). [3-NT^7/61^]CXCL12 induced significant monocyte chemotaxis after stimulation with doses of 1.25 nm and higher, with a maximal chemotactic index of 2.8 ± 0.2. At 0.375, 3.75, and 12.5 nm, [3-NT^7/61^]CXCL12 was significantly less potent at inducing chemotaxis when compared to CXCL12. THP-1 cell migration after stimulation with [3-NT^61^]CXCL12 was significantly lower compared to CXCL12 at 12.5 nM. On the other hand, a dose of 1.25 nM [3-NT^61^]CXCL12 induced a significantly higher chemotactic response compared to CXCL12. This was the result of a shift of the typical bell-shaped dose response curve after stimulation of THP-1 cells with [3-NT^61^]CXCL12 compared to the dose response curve of THP-1 cell migration toward CXCL12. As a result, the maximal chemotactic index was reached at a lower dose of [3-NT^61^]CXCL12 than of CXCL12 (3.75 and 12.5 nM, respectively). However, the maximal chemotactic response was stronger for CXCL12 than for [3-NT^61^]CXCL12 at their respective optimal dose (maximal chemotactic index of 6.2 ± 0.9 and 4 ± 0.4, respectively).

Chemotaxis of freshly isolated lymphocytes toward various concentrations of CXCL12, [3-NT^61^]CXCL12 and [3-NT^7/61^]CXCL12 was compared (Figure 10 middle panel). Both CXCL12 and [3-NT^61^]CXCL12 induced lymphocyte chemotaxis at all tested doses. [3-NT^7/61^]CXCL12 was able to induce a significant chemotactic response at all doses higher than 3.75 nM, and the maximal chemotactic index obtained was 2.7 ± 0.7. CXCL12 was significantly more potent than [3-NT^7/61^]CXCL12 in attracting lymphocytes at every applied dose, except 375 nM. *In vitro* lymphocyte chemotaxis was comparable for CXCL12 and [3-NT^61^]CXCL12 (maximal chemotactic index of 4.3 ± 0.5 for CXCL12, compared to 4.8 ± 1 for [3-NT^61^]CXCL12).

Also the effect of tyrosine nitration on the capacity of CXCL12 to induce endothelial cell migration was tested (Figure 10 lower panel). Whereas, CXCL12 significantly induced endothelial cell migration at any given dose, the other three CXCL12 forms only did so at doses of 12.5 nM and higher. CXCL12 was significantly more potent at attracting endothelial cells compared to [3-NT^7^]CXCL12 and [3-NT^7/61^]CXCL12 at 12.5 nM. No statistical differences were measured between CXCL12 and [3-NT^61^]CXCL12.

### Nitration of CXCL12 reduces its lymphocyte chemoattractant activity *in vivo*

**Figure 11:**
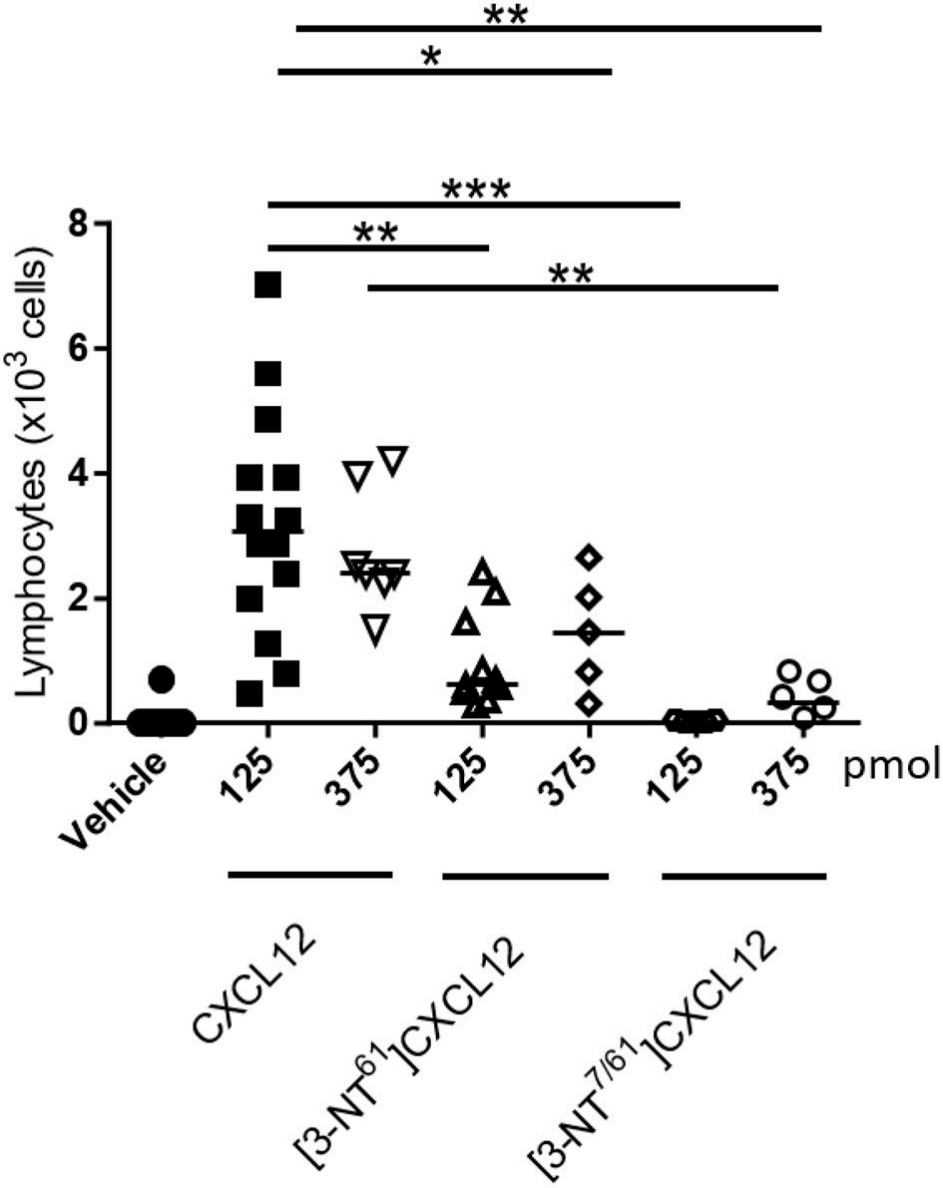
*In vivo* lymphocyte extravasation to the articular cavity following injection of native and nitrated CXCL12. Vehicle, CXCL12, [3-NT^61^]CXCL12 or [3-NT^7/61^]CXCL12 (125 and 375 pmol) were injected in 10 μl into the knee cavity of C57BL/6 mice that were treated for 48 h with the CD26 inhibitor sitagliptin (added to the drinking water). Three hours after injection, recruited leukocytes were counted differentially on May-Grünwald-Giemsa stained cytospins. Each symbol represents an individual mouse (*n* ≥ 5). The median number of migrated lymphocytes is shown as a horizontal line. Statistical analysis comparing CXCL12 with [3-NT^61^]CXCL12 and [3-NT^7/61^]CXCL12 was performed using the Mann-Whitney *U* test (**p < 0.01; ***p < 0.001).

The consequences of nitration on CXCL12-mediated lymphocyte extravasation was also investigated *in vivo*. Several doses of CXCL12, [3-NT^61^]CXCL12, and [3-NT^7/61^]CXCL12 were injected into the tibiofemoral articulation of mice, after which the recruited lymphocytes were recovered and counted (Figure 11). To protect the injected CXCL12 from proteolytic truncation by CD26 and its consequent inactivation, the mice were given drinking water containing the CD26 inhibitor sitagliptin from 48 h prior to the injection of chemokine till the end of the experiment. All tested doses, except 125 pmol [3-NT^7/61^]CXCL12, caused a significant extravasation of lymphocytes as compared to vehicle control. Injection of 125 pmol native CXCL12 resulted in the migration of a median amount of 3 × 10^3^ lymphocytes to the joint. This number was significantly higher when compared to the extravasation following the injection of 125 pmol [3-NT^7/61^]CXCL12 or [3-NT^61^]CXCL12, attracting no lymphocytes and a median number of 0.6 × 10^3^ lymphocytes, respectively. Although the injection of 375 pmol [3-NT^7/61^]CXCL12 significantly recruited lymphocytes to the knee cavity (median number of 0.4 × 10^3^ cells), the number of recruited cells was significantly lower when compared to the number of cells recruited after injection with 375 pmol CXCL12 (median number of 2.4 × 10^3^ cells). The injection of 375 pmol [3-NT^61^]CXCL12 showed a trend of reduced lymphocyte extravasation (median number of 1.4 × 10^3^ cells) compared to CXCL12. However, this difference was not statistically significant (*p* = 0.06).

### COOH-terminal nitration of CXCL12 results in a decreased sensitivity for CD26 cleavage

**Figure 12:**
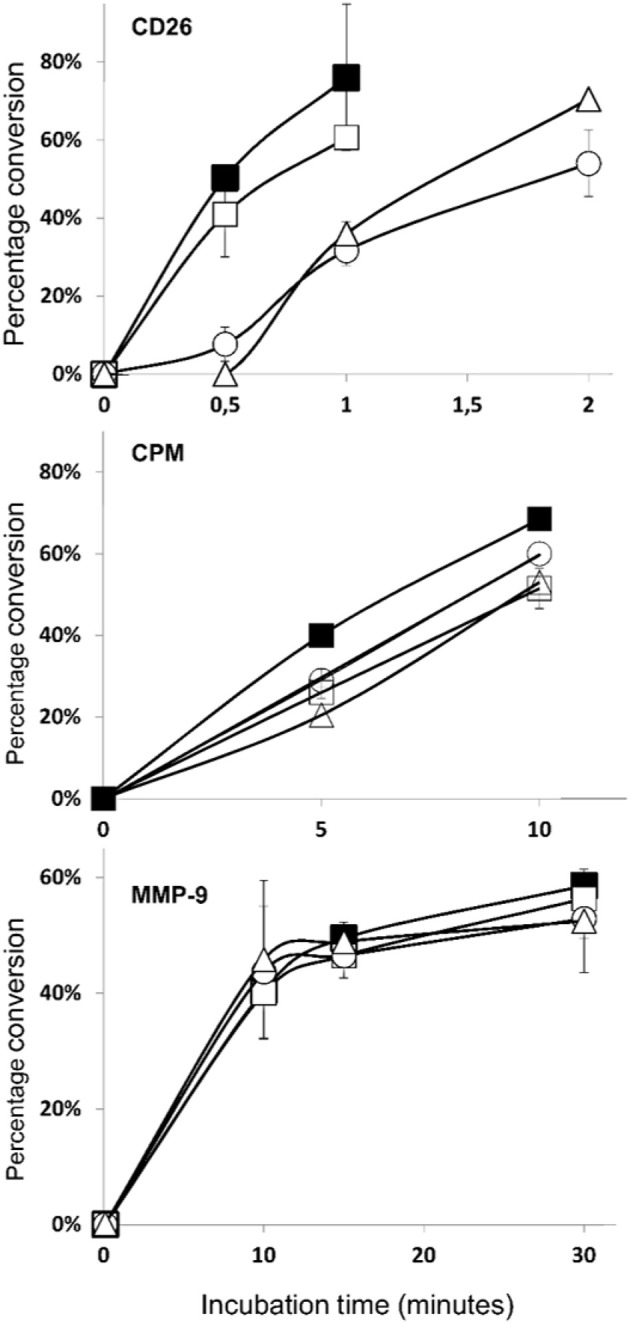
Truncation of CXCL12 and its nitrated variants by CD26, CPM, and MMP-9. A final concentration of 5 μM CXCL12 (■, filled squares), [3-NT^7^]CXCL12 (□, open squares), [3-NT^61^]CXCL12 (°, open circles) or [3-NT^7/61^]CXCL12 (Δ, open triangles) was incubated with CD26 (1.65 nM or 5 U/L) (upper panel), CPM (15 nM) (middle panel) or MMP-9 (10 nM) (lower panel) at 37°C. The incubated samples were desalted on C18 ZipTips and analyzed by mass spectrometry. The graphs show the mean percentage conversion (±SEM) of intact chemokine in function of time (*n* = 3 for CD26, *n* = 2 for CPM and MMP-9). CPM, carboxypeptidase M; MMP-9, matrix metalloproteinase-9.

As mentioned before, an important mechanism to regulate the activity of CXCL12 is enzymatic truncation of either its NH_2_- or COOH-terminus. We tested the effect of nitration on the susceptibility of CXCL12 to become enzymatically processed by three enzymes that were reported to process CXCL12, i.e., CD26, CPM, and MMP-9. Figure 12 shows the results of the incubation experiments where the conversions of CXCL12 and its nitrated variants by CD26 (upper panel), CPM (middle panel), and MMP-9 (lower panel) were measured. The half-life of the tested CXCL12 variants for these enzymes were calculated and are shown in Table 1. CXCL12 and [3-NT^7^]CXCL12 were rapidly cleaved by CD26, with a half-life of < 1 min. [3-NT^61^]CXCL12 and [3-NT^7/61^]CXCL12 were cleaved slower by CD26, with a half-life that was two to three times longer compared to the half-life of unmodified CXCL12. All tested CXCL12 variants generally showed a similar susceptibility to proteolytic cleavage by CPM or MMP-9. For CPM, the half-life varied between 7 and 9.8 min,

**Table 1:** Half-lives of CXCL12, [3-NT^7^]CXCL12, [3-NT^61^]CXCL12 and [3-NT^7/61^]CXCL12 after incubation with CD26, CPM, or MMP-9.

	**Half-life (min)**		
	**CD26**	**CPM**	**MMP-9**
CXCL12	0.6	7	16.9
[3-NT^7^]CXCL12	0.8	8.4	20.8
[3-NT^61^]CXCL12	1.8	9.7	21.2
[3-NT^7/61^]CXCL12	1.5	9.8	18.5

*CPM, carboxypeptidase M; MMP-9, matrix metalloproteinase-9*.

whereas cleavage by MMP-9 occurred slower with a half-life ranging from 17 to 21 min.

### NH_2_-terminal nitration of CXCL12 reduces its Anti-HIV-1 activity

**Figure 13:**
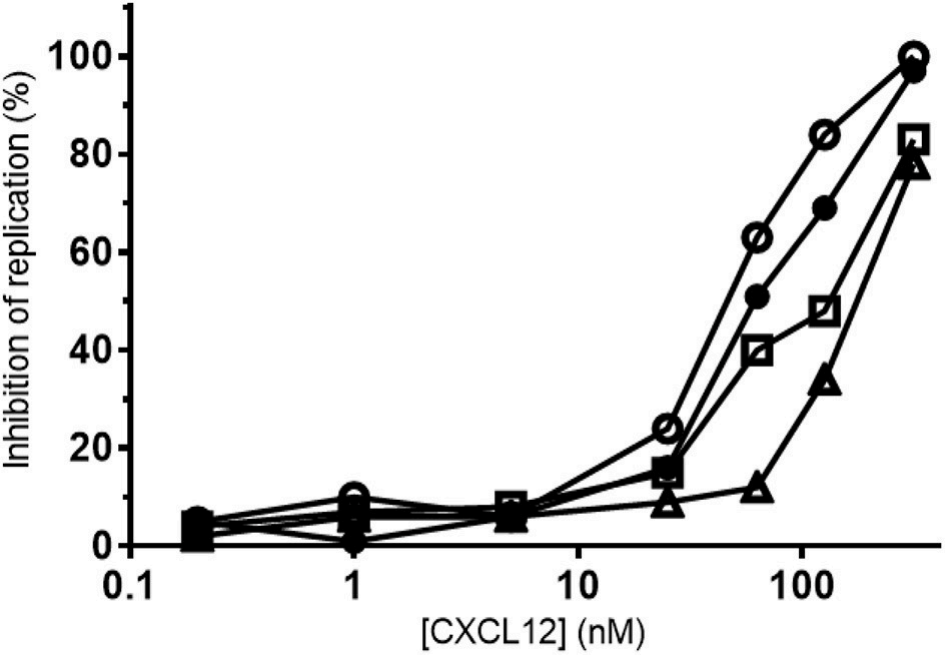
Anti-HIV-1 activity of nitrated CXCL12 forms. Serial dilutions of CXCL12, (■, filled squares), [3-NT^7^]CXCL12 (□, open squares), [3-NT^61^]CXCL12 (°, open circles) and [3-NT^7/61^]CXCL12 (Δ, open triangles) ranging from 0.2 to 375 nM were incubated with lymphocytic MT-4 cells at the moment of infection with the HIV-1 strain NL4.3. Mean percentages of the inhibition of HIV-1 replication were calculated from triplicate cultures. One representative experiment out of two is shown.

Besides CCR5, CXCR4 is a main co-receptor for HIV-1 infection. Since CXCL12 is a natural competitor for HIV-1 gp120 binding to CXCR4, the effect of tyrosine nitration on its antiviral activity was investigated on lymphocytic MT-4 cells using the CXCR4-tropic NL4.3 HIV-1 strain. A representative experiment of the inhibition of HIV-1 replication by the CXCL12 forms is shown in Figure 13. A summary of the IC_50_ values of the CXCL12 forms, calculated for two independent experiments is depicted in Table 2. The small-molecule CXCR4 inhibitor AMD3100 was used as a positive control and showed an average IC_50_ of 7.9 nM. CXCL12 inhibited HIV-1 NL4.3 replication with an average IC_50_ of 70.5 nM. [3-NT^61^]CXCL12 had a slightly better anti-HIV-1 activity, with an IC_50_ of 41 nM. In contrast, nitration of Tyr7 or both Tyr residues resulted in a lower anti-HIV-1 activity, with average IC_50_ values of 125 nM for [3-NT^7^]CXCL12 and 180 nM for [3-NT^7/61^]CXCL12.

**Table 2:** Anti-HIV activity of CXCL12 and its nitrated variants in lymphocytic MT-4 cells.

	**Experiment 1 (IC50, nM)**	**Experiment 2 (IC50, nM)**
CXCL12	87	54
[3-NT^7^]CXCL12	151	99
[3-NT^61^]CXCL12	41	41
[3-NT^7/61^]CXCL12	201	159
AMD3100	7.8	8

## Discussion

Peroxynitrite is a short-lived oxidizing reagent that is generated through the interaction of nitric oxide and superoxide anions. Production of these molecules occurs during the oxidative response of macrophages, eosinophils and neutrophils as a response to tumoral or microbial stress or during reperfusion injury (37). Due to the unstable character and therefore short half-life (<10 ms) of peroxynitrite, at physiological *pH*, a direct determination of peroxynitrite concentrations *in vivo* is difficult. Peroxynitrite concentrations are estimated to be in the nanomolar range. However, due to continuous production and release, these concentrations can be maintained for hours (38). Peroxynitrite can nitrate amino acids that contain ring structures, like Tyr and Trp (39). To study the effects of nitration on proteins, they are usually incubated for a short time with a bolus of highly concentrated peroxynitrite (concentrations ranging from 10 μM to 1 mM) (30, 40, 41). Specific nitrotyrosine antibodies have been developed and the presence of nitrotyrosine in tissues is considered as a marker of inflammation since it is the result of the coexistence of reactive nitrogen and oxygen species.

Co-localization studies showed that areas with nitrotyrosine and CCL2 immunoreactivity coincided in kidney tissue sections of ischemia and reperfusion-induced acute tubular necrosis patients (41). In tissue sections of human colon and prostate carcinoma, nitrated CCL2 was detected using nanobodies (30). *In vitro* Tyr nitration by incubation with peroxynitrite was detected for CCL2, CCL3, CCL5, CCL11, CXCL8 and CXCL12 (30, 40–45). Nitration of CXCL12 was also described to occur *in vitro* by co-culture of bone marrow cells and leukocytes when inflammatory stimuli were added to the cell medium (26). Both eosinophil chemotaxis toward CCL5 and CCL11 and migration of monocytes and neutrophils toward CCL3 were reduced after incubation of these chemokines with peroxynitrite (42–44). Regarding CCL2, Molon and colleagues showed an abolished CD8^+^ T cell migration, whereas Barker and colleagues reported only a reduced T cell chemotaxis after incubation of the chemokine with peroxynitrite (30, 41). Monocyte chemotaxis was reduced as a result of CCL2 nitration (30, 40, 41). Also the migration of lymphocytes and monocytes to CXCL12 was reduced as a consequence of Tyr nitration (26, 30).

In the published studies in which peroxynitrite was incubated with recombinant chemokines, the presence of nitrotyrosine was detected using direct ELISA assays or MS^2^ analyses of tryptic digests. The aim of the present study was to detect the appearance of nitrotyrosine in intact CXCL12 after incubation with peroxynitrite since we noticed that also complete degradation of chemokines may occur upon treatment with peroxynitrite (data not shown). We confirmed the presence of nitration on Tyr7, Tyr61, or both, but importantly not on Trp. This indicates that although not specific for one of the two Tyr amino acids, Tyr is more sensitive to modification than Trp. The percentage of nitrated CXCL12 gradually increased as the applied peroxynitrite concentrations were elevated. At 1 mM, peroxynitrite nitrated approximately 55% of the detected CXCL12. To be able to evaluate biochemical and biological characteristics, additional CXCL12 variants containing a nitration on Tyr61, i.e., [3-NT^61^]CXCL12 and [3-NT^7/61^]CXCL12, were chemically synthesized, folded and purified by reversed-phase HPLC.

To gain further knowledge on the effects of tyrosine nitration on CXCL12 activity, CXCL12, [3-NT^7^]CXCL12, [3-NT^61^]CXCL12, and [3-NT^7/61^]CXCL12 were tested in *in vitro* and *in vivo* biological assays. [3-NT^7^]CXCL12, [3-NT^61^]CXCL12, and [3-NT^7/61^]CXCL12 had a similar heparin, CXCR4 and ACKR3 binding affinity when compared to CXCL12 (26). However, remarkably, compared to CXCL12, we measured an increased affinity of [3-NT^7^]CXCL12, [3-NT^61^]CXCL12, and [3-NT^7/61^]CXCL12 for another GAG, i.e., chondroitin sulfate. For several chemokines it has been shown that binding affinities for GAGs greatly vary depending on the used GAG (46). Here, we show that post-translational nitration of CXCL12 increases its affinity for chondroitin sulfate, whereas the affinity for heparin remains similar for nitrated and native CXCL12. Internalization of CXCR4 after stimulation with [3-NT^7/61^]CXCL12 and the earlier described [3-NT^7^]CXCL12 was similar, showing a reduced potency to internalize CXCR4 at intermediate concentrations (26). Recruitment of β-arrestin 2 after stimulation of CXCR4 with [3-NT^7^]CXCL12 and [3-NT^7/61^]CXCL12 was also significantly reduced, explaining the moderately diminished receptor internalization [Figure 6 and 26). However, β-arrestin 2 recruitment to ACKR3 remained unaltered for all CXCL12 forms indicating that modification of CXCL12 has more profound effects on its signaling through CXCR4 than ACKR3 and induces at least a partial receptor bias.

In general and in contrast to nitration of Tyr7 (26), nitration of Tyr61 did not negatively influence signal transduction but even showed a moderately enhanced potency to induce phosphorylation of the second messengers Akt and ERK. [3-NT^7/61^]CXCL12 was significantly weaker in inducing an increase in intracellular calcium and phosphorylation of Akt and ERK when compared to native CXCL12. Surprisingly, nitration of CXCL12 did not result in a different potency to induce IP_3_ recruitment to the cytoplasm of COS-7 cells despite the clear effects in calcium assays on both THP-1 cells and CHO-CXCR4 transfectants. Although different cells were used in this assay and the expression levels of CXCR4 were lower on COS-7 compared to CHO cells (data not shown), we do not have a clear explanation for this since IP_3_ recruitment was evaluated at multiple concentrations and after different incubation periods. The reduced signaling potency was translated into a reduced THP-1 monocyte and lymphocyte *in vitro* chemotaxis toward [3-NT^7/61^]CXCL12 compared to CXCL12, as was previously described for the NH_2_-terminally nitrated [3-NT^7^]CXCL12 (26). Since CXCL12 is a proangiogenic factor and tyrosine nitration was detected in kidney tissues after ischemia reperfusion injury, we also tested whether endothelial cell migration toward CXCL12 was altered by tyrosine nitration (41, 47). Chemotaxis of endothelial cells following stimulation with [3-NT^7^]CXCL12 and [3-NT^7/61^]CXCL12 was significantly reduced, whereas endothelial cell migration toward [3-NT^61^]CXCL12 was similar to endothelial chemotaxis toward CXCL12.

Remarkably, whereas CXCL12 and [3-NT^61^]CXCL12 showed similar *in vitro* characteristics, 125 pmol [3-NT^61^]CXCL12 attracted significantly less lymphocytes to the tibiofemoral articulation compared to 125 pmol CXCL12. Also a non-significant trend (*p* = 0.06) of lower numbers of extravasated lymphocytes toward 375 pmol [3-NT^61^]CXCL12 compared to 375 pmol CXCL12 was observed. Similar to the previously described [3-NT^7^]CXCL12 (26), injection of [3-NT^7/61^]CXCL12 in the knee joint attracted only very low numbers of lymphocytes at any given dose. Barker and colleagues observed reduced monocyte chemotaxis in *in vivo* air pouch experiments as a result of CCL2 nitration. They attributed this effect to a reduced heparin and heparan sulfate binding capacity of the nitrated CCL2 (41). We showed that heparin binding properties of [3-NT^61^]CXCL12 and [3-NT^7/61^]CXCL12 were similar to CXCL12 in this study. Also in our previous study, [3-NT^7^]CXCL12 had similar binding potencies to heparin, heparan sulfate and dermatan sulfate (26). Previously, we also observed more profound differences *in vivo* compared to *in vitro* for other post-translationally modified chemokines. Truncated CXCL5 was 3 to 10-fold more potent than intact CXCL5 *in vitro*. However, in order to be active *in vivo*, proteolytic cleavage of CXCL5 was essential (48). Citrullinated CXCL8 was almost equally potent as unmodified CXCL8 in *in vitro* signaling and chemotaxis assays but failed to recruit neutrophils *in vivo* (49). We did not observe a difference in degradation between the CXCL12 forms upon incubation with peroxynitrite. However, the addition of a nitro group to Tyr7 or both Tyr7 and Tyr61 significantly increased the affinity of CXCL12 for chondroitin sulfate. This GAG is highly abundant in cartilage and in the used *in vivo* model, CXCL12 was injected in the knee cavity. CXCL12 or the nitrated variants are thus expected to interact with chondroitin sulfate. The *in vivo* activity of CXCL12 depends on its interaction with CXCR4, ACKR3 and GAGs. Although difficult to prove, our data suggest that the altered chondroitin sulfate interaction may at least partially explain the *in vivo* differences. The effect of GAGs on the activity of chemokines is highly complex. GAG-binding is required for *in vivo* activity (50). GAG-binding has been reported also to protect CXCL12 and CXCR3 ligands from cleavage by CD26 (51, 52). However, in our *in vivo* experiments this protease was inhibited with sitagliptin. Multiple proteases have been reported to be able to process CXCL12 (23). Although in the few investigated cases GAG-binding appears to protect chemokines from cleavage, it is unknown whether GAGs influence the sensitivity of CXCL12 to the more than 10 proteases reported to process this chemokine. Moreover, the same GAGs that protected CXCR3 ligands from cleavage by CD26 directly reduced the GPCR-dependent signaling activity of CXCR3 ligands (52). This indicates that depending on the local context, GAG-interactions may stabilize chemokines or inhibit GPCR-dependent signaling activity. In addition, the tighter interaction of the nitrated CXCL12 with chondroitin sulfate may make the NH_2_-terminus less available for receptor activation. Further investigations need to be performed to elucidate how this more thorough chondroitin sulfate binding could result in reduced *in vivo* activity.

Since proteases are known to regulate CXCL12 activity, we tested how nitration of CXCL12 influences its susceptibility to cleavage by CD26, CPM, and MMP-9. CXCL12 and [3-NT^7^]CXCL12 had a comparable half-life to previously described values (53). However, the half-life of [3-NT^61^]CXCL12 and [3-NT^7/61^]CXCL12 was two to three times longer. Thus, although CD26 cleaves a dipeptide from the NH_2_-terminus, its activity is negatively influenced by COOH-terminal nitration. As mentioned before, in our *in vivo* model we inhibited CD26 activity with sitagliptin, making it unlikely that the activity of CD26 could explain the observed reduction in CXCL12 activity *in vivo* after COOH-terminal nitration. We further confirmed that CXCL12 is processed by CPM and additionally specified that the half-life of its C-terminus under physiological conditions was ±7 min (54). Nitration of any of the tyrosine residues did not alter the susceptibility of CXCL12 to CPM-mediated processing significantly, with a half-life of the nitrated variants ranging from ±8.4 to ±9.8 min. Truncation of CXCL12 by several MMPs was discovered previously (55). We further specified that the half-life of CXCL12 for MMP-9 under physiological conditions was ±17 min and that nitration of CXCL12 did not influence its processing rate by MMP-9.

Additionally, we tested the effects of nitration on the anti-HIV-1 capacity of CXCL12. Previous studies already showed that post-translational modifications may strongly affect the ability of CXCL12 to inhibit HIV-1 infection. The enzymatic removal of the two NH_2_-terminal amino acids by CD26 drastically reduced the anti-HIV-1 potency of CXCL12 (56–58). Citrullination of Arg8 also strongly reduced the anti-HIV-1 activity of CXCL12, whereas citrullination of three or all five Arg residues completely abolished competition with HIV-1 (25). Here, [3-NT^61^]CXCL12 showed a similar competition with HIV-1 compared to CXCL12, whereas, [3-NT^7^]CXCL12 and [3-NT^7/61^]CXCL12 had a reduced anti-HIV-1 activity. Compared to NH_2_-terminal truncation or citrullination of CXCL12, NH_2_-terminal nitration only moderately reduced competition with HIV-1. Accordingly, also the effects of truncation and citrullination on receptor activation and cellular migration were more drastic compared to the effects of nitration of CXCL12. Since the CXCR4 binding affinity was similar for all tested CXCL12 forms, the lower anti-HIV-1 activity of [3-NT^7^]CXCL12 and [3-NT^7/61^]CXCL12 is probably due to the decreased capacity to induce CXCR4 internalization.

Recently, it was shown that i.v. injected nitrated CCL2 significantly inhibited cellular migration toward CCL2-containing air pouches (41). In view of this finding, the effects of CXCL12 nitration might have more implications *in vivo*. As CCL2 was shown to be nitrated after ischemia reperfusion injury, other chemokines like CXCL12 could also be nitrated in similar inflammatory events. Given its function as a stem and progenitor cell chemoattractant, migration of these cell types by circulating nitrated CXCL12 might be inhibited and thus potentially hinder tissue repair and revascularization after injury.

In conclusion, we showed that intact CXCL12 is nitrated on Tyr7 and Tyr61, but not on Trp57. Nitrated CXCL12 showed an increased chondroitin sulfate binding affinity, whereas the heparin and receptor binding potency remained comparable to native CXCL12. Post-translational nitration of Tyr7 or both Tyr7 and Tyr61 resulted in a lowered CXCR4-mediated β-arrestin 2 recruitment and subsequent CXCR4 internalization. Contrary, β-arrestin 2 recruitment to ACKR3 remained the same for all CXCL12 forms, indicating a bias toward internalization via ACKR3. Moreover, calcium signaling, *in vitro* migration of monocytes, lymphocytes and endothelial cells and anti-HIV-1 potency were reduced after NH_2_-terminal Tyr nitration. Nitration of any tyrosine residue did not significantly influence the processing speed of CXCL12 by CPM and MMP-9, whereas, remarkably, only COOH-terminal and not NH_2_-terminal nitration resulted in CXCL12 variants that were less efficiently cleaved by the aminopeptidase CD26. *In vivo*, nitration of any Tyr residue resulted in a lowered lymphocyte extravasation into the knee joint.

## Author contributions

RJ, AM, SS, and PP designed the study. RJ performed nitration experiments, synthesized the chemokine forms, performed receptor binding and internalization, calcium, Akt and ERK signal transduction and monocyte and lymphocyte chemotaxis experiments and wrote the manuscript under supervision of PP. VV performed GAG binding experiments. SN performed biacore experiments under supervision of SL. OL performed IP3 experiments and VD performed beta-arrestin recruitment experiments, both under the supervision of MR. PR performed signaling assays and endothelial cell migration experiments under supervision of SS and SL. RJ, DB, PP, FA and MT designed and performed the *in vivo* experiments. DS performed anti-HIV assays. All authors read and corrected the manuscript.

### Conflict of interest statement

The authors declare that the research was conducted in the absence of any commercial or financial relationships that could be construed as a potential conflict of interest.
